# Virulence Potential and Antibiotic Susceptibility of *S. aureus* Strains Isolated from Food Handlers

**DOI:** 10.3390/microorganisms10112155

**Published:** 2022-10-30

**Authors:** Adriana Fernandes, Carla Ramos, Victor Monteiro, Joana Santos, Paulo Fernandes

**Affiliations:** 1 Escola Superior de Tecnologia e Gestão, Instituto Politécnico de Viana do Castelo, Rua Escola Industrial e Comercial de Nun’Álvares, 4900-347 Viana do Castelo, Portugal; 2 CISAS, Escola Superior de Tecnologia e Gestão, Instituto Politécnico de Viana do Castelo, Rua Escola Industrial e Comercial de Nun’Álvares, 4900-347 Viana do Castelo, Portugal

**Keywords:** *S. aureus*, food safety, antibiotic resistance, enterotoxins, *Staphylococcal*

## Abstract

*Staphylococcus* spp. are common members of the normal human flora. However, some *Staphylococcus* species are recognised as human pathogens due to the production of several virulence factors and enterotoxins that are particularly worrisome in food poisoning. Since many of Staphylococcal food poisoning outbreaks are typically associated with cross-contamination, the detection of *S. aureus* on food handlers was performed. Hand swabs from 167 food handlers were analysed for the presence of *S. aureus*. More than 11% of the samples were positive for *S. aureus*. All *S. aureus* strains were isolated and analysed for the presence of virulence and enterotoxin genes, namely, *sea*, *seb*, *sec*, *sed*, *seg*, *sei*, *tsst*-1 and *pvl*. The same strains were phenotypically characterised in terms of antibiotic susceptibility using the disc diffusion method and antimicrobial agents from 12 different classes. A low prevalence of antibiotic-resistant strains was found, with 55.6% of the strains being sensitive to all of the antimicrobial agents tested. However, a high prevalence of resistance to macrolides was found, with 44.4% of the strains showing resistance to erythromycin. At least one of the virulence or toxin genes was detected in 61.1% of the strains, and *seg* was the most prevalent toxin gene, being detected in 44.4% of the strains.

## 1. Introduction

A high number of foodborne outbreaks are associated with the wrong practices and poor hygiene of food handlers [1]. According to the “The European Union One Health 2019 Zoonoses Report” [2], a total of 3086 foodborne outbreaks were reported by 27 member states, and among outbreaks with known causative agents, most were due to bacteria, followed by bacterial toxins. Among the bacterial toxins reported by 13 member states, 1.4% were caused by *Staphylococcus* spp., including both strong-evidence and weak-evidence outbreaks. These were reported by eleven member states, and staphylococcal enterotoxins were found in samples such as cheeses, salads, cakes, egg products and other processed foods and prepared dishes [2]. In previous years, such as 2015, in Europe, a significant number of the total foodborne outbreaks were caused by staphylococcal toxins, and most of the reported places of exposure for strong-evidence outbreaks were a household or “restaurant, café, bar, hotel or catering service”. An “infected food handler” was reported as a contributory factor in many of these outbreaks [3]. According to Greig, Todd et al. [4], and citing CDC, there is an estimation that approximately 20% of outbreaks of foodborne diseases are due to contamination by infected food handlers. This situation is often explained by the incorrect handling of already-cooked food or storage at improper temperatures, allowing for *S. aureus* growth and toxin production. In fact, the human body is a natural reservoir of many microorganisms, among which *Staphylococcus* spp. are very common (20–60%) on the skin and in the nasal cavities of healthy individuals [5,6]. Food contamination can therefore be easily caused by food handlers due to the direct contact of processed food with hands or surfaces or even due to sneezing.

The possibility of the transmission of these microorganisms between food handlers, surfaces and food is a problem affecting the food industry [7], contributing to frequent foodborne outbreaks. Also relevant is that antimicrobial-resistant strains are spreading in the environment and community and are no longer restricted to healthcare environments. In the last two decades, the emergence and transmission of multidrug-resistant pathogenic bacteria have created an urgent need to understand the underlying mechanisms involved [8] in the spread of these biological agents. Methicillin-resistant *S. aureus* (MRSA) strains emerged after the introduction of methicillin and are now an important public health problem [9].

Several virulence factors are associated with the pathogenicity of *S. aureus* strains, including the production of extracellular enzymes, such as lipases, proteases, amylases, hyaluronidase, DNases, coagulase and lactamase; the production of several toxins and enterotoxins, such as β, ɣ and δ haemolysins; and also the production of surface components, such as the capsule [10,11,12,13,14].

Since many of the Staphylococcal food poisoning outbreaks are typically associated with cross-contamination due to the handling of food, the detection of *S. aureus* on the hands of food handlers was performed. The main objective of this work was to determine the prevalence of *S. aureus* associated with food handlers in their work environment, as well as to analyse the potential virulence, toxicity and antibiotic resistance of the isolated strains.

The isolated strains were therefore analysed for the presence of several enterotoxin genes (*sea*, *seb*, *sec*, *sed* and *seg*), toxic shock syndrome toxin, pvl, and mecA and mecC, the main genes conferring resistance to methicillin. Phenotypic studies on strain resistance to antibiotics are also presented, as well as the biochemical characterisation of the isolates with respect to the production of enzymes capable of compromising the quality of food, such as proteases, lipases and amylases.

## 2. Materials and Methods

### 2.1. Sample Collection and Processing

The collection of hand swab samples was performed on previously informed food handlers who voluntarily consented to participate in establishments in the district of Viana do Castelo in the North of Portugal as part of routine hygiene control. Samples, collected for a period of 13 months, were transported to the laboratory in 15 mL Falcon tubes containing transport medium (30 g/L Tween 80 (Fisher Chemical (Mt Prospect, IL, USA), 3 g/L lecithin (Prolabo BDH, Geldenaaksebaan, Belgium), 5 g/L sodium thiosulfate (Panreac Applichem, Darmstadt, Germany), 1 g/L L-histidine (Merck, Darmstadt, Germany) and 30 g/L saponin (Prolabo BDH, Geldenaaksebaan, Belgium)). The transport was performed in cooled insulated containers (1–8 °C). After their reception at the laboratory, samples were kept at 3 ± 2 °C and processed within a maximum of 24 h from sampling. The same amount of sample (in duplicate) was mixed with 2× concentrated Buttiaux-Brogniart Hypersalted Broth (Biokar Diagnostics, Beauvais, France), incubated at 37 ± 1 °C (Sanyo Incubator, Japan) for 48 h and then surface-inoculated on Bair Parker Agar (Biokar Diagnostics, Beauvais, France). After incubation for 24–48 h 37 ± 1 °C, typical colonies were transferred to BHI medium (Biokar Diagnostics, Beauvais, France) and incubated for 20–24 h at 37 °C. The confirmation of coagulase-positive *Staphylococcus* was performed by transferring 0.1 mL of the BHI culture to 300 mL of rabbit plasma Biokar Diagnostics, Beauvais, France. After incubation at 37 °C for 4–6 h, coagulation was evaluated.

### 2.2. Storage and Regeneration of Bacterial Cultures

Eighteen strains isolated from hand swabs and previously identified as coagulase-positive *Staphylococcus* were preserved at −80 °C in BHI medium with 15% (*v*/*v*) glycerol (Merck, Darmstadt, Germany) and regenerated in 5 mL of BHI medium at 37 °C overnight.

### 2.3. DNA Extraction

After the regeneration of *S. aureus* cultures, DNA extraction was performed using the Wizard^®^ Genomic DNA Purification kit (Promega, Madison, WI, USA). Extraction was carried out according to the manufacturer’s instructions with some modifications. Cells were pre-treated with 70% ethanol (Fisher Chemical, Cambridge, UK) according to the procedure described by Kalia et al. (1999).

Cells were further incubated at 37 °C for 60 min after the addition of 100 μL of 40 mg/mL lysozyme (Sigma-Aldrich GmbH, Steinheim, Germany) to facilitate lysis.

Isolated DNA was analysed by electrophoresis and kept at −20 °C until used. Using the same method described above, DNA from the control strains (*S. aureus* NCTC 6571 (*seg*^+^, *sei*^+^, *pvl*^+^), *S. aureus* ATCC 33591 (*mecA*^+^, *pvl*^−^), *S. aureus* NCTC 10654 (*seb*^+^), *S. aureus* NCTC 10652 *(sea*^+^, *sed*^+^) and *S. aureus* NCTC 11963 *(Tsst*-1^+^, *sec*^+^)) was also extracted.

### 2.4. S. aureus Genotyping

The 18 strains of *S. aureus* were submitted to genotypic *spa* typing by RT-PCR using the primers spa1113F and spa1514R F [15]. The amplification reactions of the DNA were performed on a CFX96 thermocycler (BioRad Inc., Hercules, FL, USA) with a total volume of 50 μL per reaction containing 5 μL of DNA from each strain, 200 nM forward primer spa1113F (5’-TAAAGACGATCCTTCGGTGAGC-3’) (STAB VIDA Lda., Lisboa, Portugal), 200 nM reverse primer 1492R (5’-CAGCAGTAGTGCCGTTTGCTT-3’) (STAB VIDA Lda., Lisboa, Portugal) and 1x iTaq™ Universal SYBR^®^ Green Supermix (2×) (BioRad Inc., Hercules, USA). The amplification was conducted under the following conditions: initial denaturation at 95 °C for 5 min, followed by denaturation for 35 cycles at 95 °C for 15 s, annealing at 60 °C for 45 s and extension at 72 °C for 90 s.

The amplified products were purified (QIAquick PCR Purification Kit, Hilden, Germany) and Sanger-sequenced at STAB VIDA Lda. (Lisboa, Portugal). The resulting chromatograms were analysed using the UGENE program (version 38.1, Unipro, Novosibirsk, Russia) for the extraction of high-quality fragments, which were further processed using DNAGear software [16].

### 2.5. Detection of Toxin Genes, Virulence Factors and Methicillin Resistance by RT-PCR

DNA amplification reactions containing 200 nM of each primer (STAB VIDA, Portugal), 8 μL of SsoFast EvaGreen^®^ Supermix dNTP (Bio-Rad, Hercules, CA, USA) and 5 μL of DNA from each strain, for a total volume of 16 μL, were performed on RT-PCR equipment (Bio-Rad CFX96 ™ Real-time System) under the following conditions: initial denaturation at 95 °C for 5 min, followed by denaturation for 30 cycles of 15 s at 95 °C. Different annealing temperatures were used over 30 cycles, namely, 30 s at 50 °C for the detection of the *sea*, *seb*, *sec* and *sed* genes; 30 s at 55 °C for the detection of the *mec*A, *mec*C and *sec* genes; and 58 °C for 1 min for the detection of the *Tsst*1 gene. The primers used for each PCR reaction are described in Table 1.

After the ligation of the primers, extension occurred at 72 °C for 30 s. One final step of extension occurred at 72 °C for 10 min. Amplification reactions were performed using control DNA from the reference cultures indicated in Section 2.3.

### 2.6. Electrophoretic Analysis of PCR Products

After amplification, 10 μL of each PCR product was mixed with 2 μL of 6× concentrated sample buffer: 10 mM Tris (Merck, Darmstadt, Germany), 500 mM EDTA (Sigma, St. Louis, MO, USA), glycerol (Scharlau, Scharlab S.L., Barcelona, Spain), 0.03% bromophenol blue Sigma) and xylene cyanol FF 0.05% (Sigma, St. Louis, MO, USA).

PCR products were analysed on 2% agarose (Sigma, St. Louis, MO, USA) gel in 0.5× TBE buffer (composed of 10 mM Tris (Merck, Darmstadt, Germany), boric acid (Sigma, St. Louis, MO, USA), 500 mM EDTA (Sigma, St. Louis, MO, USA) and distilled water) with 0.5 μL/mL ethidium bromide (Sigma, St. Louis, MO, USA).

As a molecular weight standard, a 5 μL DNA ladder (Cleaver Scientific, Rugby, UK) was used on the same agarose gel.

Electrophoresis was performed on horizontal electrophoresis equipment (Horizon^®^ 58, Life Technologies, Carlsbad, CA, USA) for 1 h and 45 min at a constant 70 Volts. Subsequently, the gel was visualised by UV illumination (Uvitec, Cambridge, UK), in which the migration of the DNA ladder bands was compared with the migration of the PCR products from the 18 samples and respective controls.

### 2.7. Preparation of Culture Medium and Inoculum Suspension for the Antibiotic Susceptibility Tests

Cultures of *S. aureus* preserved at −80 °C were regenerated in 5 mL of BHI medium at 37 °C overnight. Subsequently, with a sterile loop, they were spiked onto TSA (Biokar Diagnostics, Beauvais, France) plates and incubated for 16–24 h at 37 °C.

Colonies of similar morphological appearance were withdrawn from the colonies isolated in TSA and resuspended in a sterile 0.85% (*w*/*v*) NaCl solution. After homogenisation, the concentration of the suspension was adjusted to 0.5 MacFarland optical density (Biosan DEN-1B, Riga, Latvia). A standard solution of 0.5 MacFarland (Biosan, Riga, Latvia) was used for the calibration of the equipment.

### 2.8. Antibiotic Susceptibility Testing by Disc Diffusion Method

Antibiotic discs (Oxoid, Thermo Scientific, Leicestershire, UK) stored at −20 °C were removed from the freezer and placed at room temperature 1 h before use.

With a sterile swab, Mueller–Hinton plates (Oxoid, Thermo Scientific, Leicestershire, UK) were inoculated with the previously prepared inoculum so as to fill the entire surface of the Petri dish (diameter 90 mm).

The discs were applied on the surface of the Mueller–Hinton medium within 15 min after the inoculation of the plates with the aid of sterile forceps at least 1.5 cm apart from each other. The plates were placed on a dark non-reflective surface, and inhibition halos were measured after 18 ± 2 h of incubation at 35 °C and interpreted according to the EUCAST guide to characterise the antimicrobial resistance of the strains of *S. aureus* [22]. A total of 14 antibiotics were used, namely, fusidic acid (10 µg), cefoxitin (30 µg), ceftaroline (5 µg), ciprofloxacin (5 µg), clindamycin (2 µg), chloramphenicol (30 µg), erythromycin (15 µg), gentamicin (10 µg), linezolid (10 µg), quinupristin–dalfopristin (15 µg), rifampicin (5 µg), teicoplanin (30 µg), tetracycline (30 µg) and tigecycline (15 µg). Inducible resistance to clindamycin was tested in all erythromycin-resistant strains by using the D-test [23] on Mueller–Hinton agar.

### 2.9. Determination of Vancomycin Resistance by Dilution (MIC)

BHI agar medium was prepared according to the manufacturer’s instructions and autoclaved at 121 °C for 15 min. The medium was allowed to cool to about 60 °C, and vancomycin (Duchefa Biochemie, Haarlem, The Netherlands) at a concentration of 6 μg/mL was added. After homogenisation, the medium was poured into Petri dishes (diameter 90 mm). The inoculum was prepared by the direct suspension of 4 or 5 morphologically similar colonies, isolated in TSA medium, in a sterile 0.85% (*w*/*v*) NaCl solution until a turbidity of 0.5 MacFarland was obtained (Biosan DEN-1B, Riga, Latvia). The previously prepared suspension was used to inoculate 10 μL spots on the BHI agar medium with vancomycin. The inoculated plates remained at room temperature until the inocula were absorbed by the medium. The plates were then inverted and incubated at 37 °C for 24 h.

The minimum inhibitory concentration (MIC) was recorded as the lowest concentration of the antimicrobial agent that completely inhibited growth, excluding the slight turbidity caused by the inoculum. Strains from the UMA collection, namely, B1037, B1061 and B1062, were used as positive controls for vancomycin resistance.

### 2.10. Biochemical Tests

#### 2.10.1. Detection of DNase-Producing Strains

Plates were prepared with agarose medium consisting of tryptose (Oxoid, Thermo Scientific, Leicestershire, UK), deoxyribonucleic acid (Sigma, St. Louis, MO, USA), sodium chloride (VWR Chemicals, Monroeville, PA, USA) and agar. The pH of the medium was set at 7.3 ± 0.2 and autoclaved at 121 °C for 15 min.

The method was based on a previously published study [24]. From the *S. aureus* colonies grown in TSA medium at 37 °C overnight, each of the 18 strains was inoculated with a sterile loop on the surface of DNase agar. The plates were inverted and incubated at 37 °C for 18–24 h. After incubation, 1N of HCL was added to the surfaces of the plates.

*S. aureus* ATCC 25923 and *S. saprophyticus* ATCC 15305 were used as positive and negative controls, respectively.

#### 2.10.2. Detection of Strains with Proteolytic Activity

For the detection of the presence of proteases, AN (Biokar Diagnostics, Beauvais, France) medium was prepared with lean milk powder (Molico^®^ Nestlé, Portugal) and 0.2 M sodium phosphate buffer (Pronolab, Portugal) at pH 7.0. The three components were autoclaved separately at 121 °C for 15 min. Under sterile conditions, the media were mixed and poured into Petri dishes (diameter 90 mm). *S. aureus* colonies were inoculated into the AN medium with a sterile loop from colonies isolated in TSA at 37 °C (Sanyo Incubator) overnight. The plates were inverted and incubated at 37 °C for up to 48 h. After incubation, a 10% trichloroacetic acid (TCA) solution (Panreac Applichem, Darmstadt, Germany) was added, and proteolytic activity was considered to be present in cultures that showed a clear zone around bacterial growth [25]. *S. aureus* NCTC 6571 and *S. saprophyticus* ATCC 15305 were used as positive and negative controls, respectively.

#### 2.10.3. Haemolysis on Blood Agar

The haemolytic assay was performed on 5% (*v*/*v*) sheep blood (COS) blood agar plates (Biomérieux, Marcy-l’Étoile, France). The inoculation of the strains of *S. aureus* to be tested was performed by spreading them on the surfaces of the plates with a sterile loop from colonies previously isolated in TSA at 37 °C overnight. The plates were inverted and incubated at 37 °C for 24–48 h.

The presence of yellow-orange colonies and clear zones around the bacterial growth was interpreted as β-haemolysis. The absence of transparent zones was considered haemolysis-negative. Transparent and greenish zones around the colonies were interpreted as partial haemolysis: α-haemolysis. *Bacillus cereus* ATCC 11778 and *S. saprophyticus* ATCC 15305 were used as positive and negative controls, respectively.

#### 2.10.4. Starch Hydrolysis

For starch hydrolysis analysis, AN medium with 0.25% (*w*/*v*) soluble starch was prepared. *S. aureus* colonies were inoculated into AN medium with a sterile loop, and after incubation for 24–48 h at 37 °C, a Lugol solution was added to the surfaces of the plates. Cultures presenting a clear halo around the colonies were considered amylase-positive.

## 3. Results and Discussion

### 3.1. S. aureus—Prevalence on Food Handlers

From 167 hand swabs taken from food handlers (restaurants and catering), a total of 18 *Staphylococcus* strains were isolated, confirmed as coagulase-positive and genotypically confirmed as *S. aureus*. These results are not surprising since the human body is a reservoir for numerous microorganisms, such as *S. aureus*. This is a ubiquitous microorganism that frequently colonises the skin of the human body (one of the main ecological niches for this species) as well as the mucous membranes, particularly in the nasal cavity [26], which is one of the most common transport sites for this species [27]. This bacterial species is present in a high proportion of healthy individuals, and it is estimated that *S. aureus* is persistent in about 20% of the general population, while 60% are intermittent carriers of this species [5]. Its transfer to food through hands during the food handling process and through the observance of poor hygiene practices is possible and, unfortunately, a common cause of cross-contamination in the food sector [1,28,29]. In addition to manual contact, respiratory secretions are also a source of food contamination by handlers [30]. The *S. aureus* strains isolated belong to 12 different *spa* types (Table 2) according to the results of the molecular typing of isolated strains, with t148 being the one occurring the most frequently (four times), followed by type t571, which occurred three times. All of these strains were found to be associated with food handlers from different establishments. Two operators from the same establishment had t267 *S. aureus,* indicating potential cross-contamination between them.

Contamination with this bacterium can also result from the contact of air, dust and surfaces with food [6], resulting in their growth and production of enterotoxins. However, as can be observed in published studies, contamination by food handlers is a frequent occurrence, given the high rate of *S. aureus* on human skin and in mucous membranes. The carriage of *S. aureus* by food handlers was recently extensively reviewed by Bencardino D. et al., and although a significant prevalence was found in the many previously reported studies, the fact is that many of them were performed using hand swabs, which, due to the common use of disinfectants, could lead to the belief that the use of nasal swabs instead could increase the degree of prevalence of *S. aureus* carriers found among handlers in the food sector [31].

### 3.2. Detection of Enterotoxin Genes

Enterotoxins are small proteins of variable size, ranging from 22 to 28 kDa [6], belonging to a family of superantigens [32,33] that cause the excessive production of cytokines due to non-specific T-cell activation [34]. *S. aureus* can produce a wide variety of enterotoxins, normally designated by letters, such as enterotoxins A, B, C, D, E, G, H, I, J, K, L, M, N, O, P, Q and R, among others [35]. The most common food poisoning outbreaks involve enterotoxins of types A, B, C and D [6,26], and each of the different enterotoxins has a specific structure and mode of action [6,32]. Staphylococcal enterotoxin A, whose structural gene is the *sea* gene, has been reported by some authors to be the most frequently involved in staphylococcal foodborne diseases, followed by enterotoxin B [32,36]. However, more recently, the prevalence of non-classical staphylococcal enterotoxin genes, such as *seg* and *sei*, has been also reported [37] in *S. aureus* isolated from food but also from food handlers [38].

**Figure 1:**
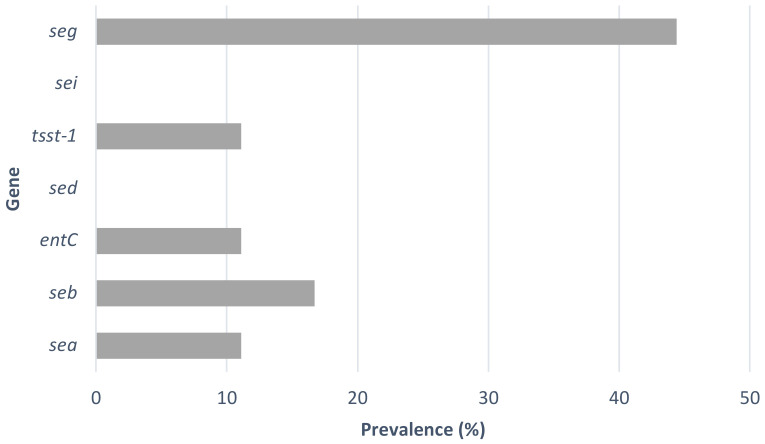
Prevalence of toxin genes in the *S. aureus* isolates.

The prevalence of toxin genes was analysed by PCR (Table 2). The genetic patterns found were: *sea/seb; tsst-1; seg; sec; seb/seg; sec/seg;* and *sea/seb/tsst-1/seg*. At least one toxin gene was present in 61.1% of the strains, whereas the *seg* gene was the most prevalent gene and could be detected in 44.4% of the strains tested (Figure 1). Some strains (11.1%) harbour two different toxin genes. The *sea*, *seb*, *tsst-1* and *seg* genes were detected in one of the strains, *S. aureus* B929, a *spa* t2540 strain (Table 2). The high prevalence of the *seg* gene in *S. aureus* strains has been previously reported. Ricardo Oliveira et al. [37] also found a prevalence of 41.9% of this enterotoxin gene in raw milk samples collected in Northern Portugal. Also in the North of Portugal, Pereira, V. et al. [39] found a high prevalence of the *seg* gene in *S. aureus* strains isolated from different food products.

The high prevalence among humans and the high rate of strains having enterotoxin structural genes could lead to the belief that the number of food Staphylococcal food poisoning outbreaks due to *S. aureus* is lower than expected. However, symptoms such as nausea, abdominal cramps, diarrhoea, vomiting, muscle pain and moderate fever, occurring between 1 and 6 h after the ingestion of contaminated food [40,41,42,43], usually do not last more than a few hours to 1–2 days. Although *S. aureus* is a common and relatively frequent cause of food poisoning, it appears, in statistical terms, to be under-reported in relation to the actual occurrence, since the symptoms usually persist for a short time, depending on the toxic dose ingested [40,43].

*Staphylococcus* species are easily destroyed by pasteurisation or normal cooking/boiling [43], but *S. aureus* enterotoxins are resistant to treatment conditions, such as heat and low pH, consequently maintaining their activity in the digestive tract after ingestion [6]. Even if the food is subjected to heat treatment, the toxins may remain active, as seen in a food outbreak caused by powdered milk and chocolate milk, in which the growth and production of enterotoxins occurred in raw milk, but the enterotoxin survived the subsequent pasteurisation process [26]. The resistance of enterotoxins to high temperatures poses additional challenges in the control of *S. aureus* outbreaks, making the adoption of good hygiene practices the best way to control this hazard.

### 3.3. Antimicrobial Susceptibility

*S. aureus* is associated with nosocomial and community-acquired staphylococcal infections, most of which are related to the emergence of antibiotic-resistant strains. In the community, most *S. aureus* infections include skin and soft tissue infections and are often caused by methicillin-resistant *Staphylococcus aureus* (MRSA) [44,45]. Since 1991, there have been increasing reports of community-acquired MRSA infections, even in individuals without risk factors for acquiring MRSA infection [46]. Infection with MRSA strains is a major cause of mortality when compared to other infections caused by *S. aureus* [47]. From these infections, dissemination can occur, leading to infections with a lethality risk and serious complications, such as endocarditis, osteomyelitis, arthritis, pneumonia, pyelonephritis or meningitis [48].

In our work, all *S. aureus* isolates were tested for antimicrobial susceptibility. A total of fifteen antimicrobial agents from twelve different antimicrobial classes were used. More than 55% of the strains tested were resistant to all of the antimicrobial agents used (Supplementary Table S1). The only antimicrobial agent to which some strains were resistant was erythromycin (Table 2). In fact, a high prevalence of resistance to erythromycin was found, as 44.4% of the tested strains presented resistance to this macrolide antibiotic. All of the resistant strains were tested for clindamycin induction by disc diffusion [23]. Except for the *S. aureus* B938 and *S. aureus* B1198 strains, which presented a D+ phenotype, all of the strains had a D phenotype, showing clear positive induction tests. In half of the resistant strains, none of the toxin or virulence genes tested were detected. However, the *seg* gene was detected in 50% of them.

Infections with antimicrobial-resistant bacteria, including methicillin resistance, can be difficult to treat [40], and MRSA strains that emerged after the introduction of this antibiotic quickly became a major public health problem [9], resulting in treatment failure and a difficult choice of alternative and effective antimicrobial agents [49]. In some cases, the only antibiotics available to effectively treat methicillin-resistant *S. aureus* infections are glycopeptides such as vancomycin [40], to which all of the strains were found to be sensitive. Erythromycin is an antibiotic used against infections caused by *S. aureus*, and the rate of erythromycin resistance we found is, in fact, very high. As early as 2012, in Poland, 60% of [50] nosocomial strains isolated from different materials were erythromycin-resistant. However, the extensive use of macrolide antibiotics leads naturally to increasing resistance to antibiotics of this class. Although several mechanisms may occur and explain the resistance to macrolides, it has been proposed that the resistance to erythromycin could be caused by a decrease in the uptake of the antibiotic into the cells [50]. The literature provides evidence of a high rate of *S. aureus* presenting antibiotic resistance genes and even multidrug resistance, with acquired resistance genes widely shared between different animal species [51], but the most prevalent resistant phenotypes are variable depending on the origin of the strains. Strains phenotypically resistant to methicillin were not found, and this is in accordance with the fact that the presence of the *mecA* gene and its homolog *mecC* was not detected in any of the *S. aureus* strains. These genes can be found in the Staphylococcal cassette chromosome *mec* (SCC*mec*) and are present in MRSA strains [52].

### 3.4. Virulence Factors

The pathogenicity of *S. aureus* is due to numerous virulence factors associated with this microorganism. Such virulence factors are diverse in nature and include surface components and enzymes such as lipases, proteases, amylases, deoxyribonucleases, coagulase and many others. *S. aureus* is also able to produce several toxins, including Leucocidin, enterotoxins and cytolytic toxins that induce erythrocyte lysis by haemolysins α, β, ɣ and δ [10,11,12,13,53]. Two-thirds of the tested strains were β-haemolytic, and all of them presented DNase activity, displaying the ability to degrade nucleic acids, as expected for *S. aureus* strains. This nuclease activity seems to increase pathogenesis by increasing the resistance of *S. aureus* to the activity of neutrophils [54]. *S. aureus* strains are also able to promote double-strand breaks in the DNA of host cells by stimulating the production of reactive oxygen species [55]. α-Haemolysis was detected in 33.3% of the strains, one of which displayed four toxin genes. The α-haemolysin protein structure seems to favour its role in the formation of a membrane-bound heptameric pore affecting the leakage of cytoplasmic content and leading to the potential death of susceptible cells [56]. All of the above factors are considered to contribute to strain virulence and hence increased pathogenicity, but in the food sector, some of these activities might also have some relevance in terms of food stability and deterioration. The expression of extracellular proteolytic enzymes with multiple roles in pathogenesis, causing either host damage or microbial adaptation, is also a characteristic of some *S. aureus* strains [57]. A serine protease, a cysteine protease, staphopain and aureolysin (a metalloprotease) are the most relevant ones [58] involved in decreasing the susceptibility to the host immune system [59]. In this sense, it is also relevant that some strains (14.4%) had clear proteolytic activity, all of them displaying resistance to erythromycin. Another virulence factor found in some *S. aureus* strains is α-amylase. This enzyme seems to be important in the adhesion process leading to the formation of biofilms [60], which are particularly important in the food sector but also relevant in pathogenesis. However, in the presented work, none of the strains tested showed activity in relation to starch hydrolysis. The gene for the cytotoxin Panton–Valentine Leucocidin (PVL), an exotoxin responsible for highly severe infections, such as in the development of abscesses and necrotizing pneumonia [61,62], was not detected in any of the *S. aureus* strains isolated.

## 4. Conclusions

Among *Staphylococcus* species, a ubiquitous and common human commensal bacterium whose habitat includes human skin, *S. aureus* is an opportunistic agent responsible for frequent nosocomial and community infections. The high incidence of this biological agent on the hands of food handlers, together with the fact that it is a bacterium with relevant pathogenic potential given the high frequency of strains with toxigenic and many other virulence factors, as well as worrisome antibiotic resistance, makes the control of the spread of this bacterium crucial to safeguarding public health. We found that this microorganism was present on more than 11% of hand swab samples from food handlers, and the isolated *S. aureus* strains had pathogenic potential, as shown by the fact that more than 60% of them had at least one enterotoxin gene. It is also noteworthy that more than 44% of the strains were resistant to erythromycin, a macrolide antibiotic frequently used to treat *S. aureus* infections. The adoption of good hygiene practices by food handlers, together with effective laboratory-based epidemiological monitoring, is thus relevant to help in decreasing the spread of *S. aureus* through cross-contamination and staphylococcal foodborne poisoning.

## Figures and Tables

**Table 1:** Primers used for the detection of toxin, virulence and antibiotic resistance genes.

Primer Int. Ref.		Obs.	
SA-U	5′-TGTATGTATGGAGGTGTAAC-3′	Forward primer used to amplify toxin genes together with primers SA-A, SA-B, SA-C, ENTC, SA-D and SA-E	[17]
SA-A	5′-ATTAACCGAAGGTTCTGT-3′	Reverse primer for *sea* gene	
SA-B	5′-ATAGTGACGAGTTAGGTA-3′	Reverse primer for *seb* gene	
SA-C	5′-AAGTACATTTTGTAAGTTCC-3′	Reverse primer for *sec* gene	
ENT-C	5′-AATTGTGTTTCTTTTATTTTCATAA-3′	Reverse primer for *sec* gene	
SA-D	5′-TTCGGGAAAATCACCCTTAA-3′	Reverse primer for *sed* gene	
SA-E	5′-GCCAAAGCTGTCTGAG-3′	Reverse primer for *see* gene	
SA-tst1-R	5′-GGCAGCATCAGCCTTATAATTT-3′	Reverse primer for *tst1* gene	[18]
SA-tst1-F	5′-GTGGATCCGTCATTCATTGTT-3′	Forward primer for *tst1 gene*	
SEG-1	5′-AAGTAGACATTTTTGGCGTTCC-3′	Forward primer for *see* gene	[19]
SEG-2	5′-AGAACCATCAAACTCGTATAGC-3′	Reverse primer for *see* gene	
SEI-1	5′-GGTGATATTGGTGTAGGTAAC-3′	Forward primer for *see* gene	
SEI-2	5′-ATCCATATTCTTTGCCTTTACCAG-3′	Reverse primer for *see* gene	
mecA-1	5′-AAAATCGATGGTAAAGGTTGGC-3′	Forward primer for *see* gene	[20]
mecA-2	5′-AGTTCTGCAGTACCGGATTTGC-3′	Reverse primer for *see* gene	
mecC-F	5′-GAAAAAAAGGCTTAGAACGCCTC-3′	Forward primer for *mecC* gene	[21]
mecC-R	5′-GAAGATCTTTTCCGTTTTCAGC-3′	Reverse primer for *mecC* gene	
pvl-F	5’-GCTGGACAAAACTTCTTGGAATAT-3’	Forward primer for *pvl* gene	
pvl-R	5’-GATAGGACACCAATAAATTCTGGATTG-3’	Reverse primer for *pvl* gene	

**Table 2:** Characterisation of the *S. aureus* isolates.

Strain Code	*spa* Type	Haemolysis	DNase Activity	Starch Hydrolysis	Proteolytic Activity	Toxin Genes Detected							Antibiotic Resistance
						*sea*	*seb*	*sec*	*sed*	*tsst-*1	*sei*	*seg*	
B863	t267	β	+	-	-								-
B864	t267	β	+	-	-					x			-
B865	t571	β	+	-	+								Erythromycin
B870	t162	β	+	-	-							x	-
B904	t530	β	+	-	-			x					-
B928	t148	β	+	-	-		x					x	-
B929	t2540	α	+	-	-	x	x			x		x	-
B937	t571	β	+	-	+								Erythromycin
B938	t130	β	+	-	-							x	Erythromycin
B939	t359	α	+	-	-								-
B1014	t084	α	+	-	-								-
B1198	t002	β	+	-	-							x	Erythromycin
B1207	t2164	β	+	-	-								Erythromycin
B1209	t148	β	+	-	-			x				x	-
B1252	t127	α	+	-	-	x	x						-
B1258	t571	β	+	-	+								Erythromycin
B1265	t148	α	+	-	-							x	Erythromycin
B1270	t148	α	+	-	-							x	Erythromycin

## Supplementary Materials

The following supporting information can be downloaded at: https://www.mdpi.com/article/10.3390/microorganisms10112155/s1, Table S1: Susceptibility of the *S. aureus* isolates to antimicrobial agents.
