# Supplementary material for: Virulence Potential and Antibiotic Susceptibility of S. aureus Strains Isolated from Food Handlers
Source: Microorganisms. 2022 Oct 30;10(11):2155. doi: 10.3390/microorganisms10112155 (PMC9696720; doi:10.3390/microorganisms10112155)
Supplement: Supplementary file 1 [file microorganisms-10-02155-s001.zip › microorganisms-2002290-supplementary.pdf]

Table S1. Susceptibility of the *S. aureus* isolates to antimicrobial agents

	Antimicrobial agent														
**Strain**	FOX	CPT	CIP	CN	TEC	E	TE	TGC	DA	QD	C	FD	LZD	RD	VA
B863	S	S	S	S	S	S	S	S	S	S	S	S	S	S	S
B864	S	S	S	S	S	S	S	S	S	S	S	S	S	S	S
B865	S	S	S	S	S	R	S	S	D	S	S	S	S	S	S
B870	S	S	S	S	S	S	S	S	S	S	S	S	S	S	S
B904	S	S	S	S	S	S	S	S	S	S	S	S	S	S	S
B928	S	S	S	S	S	S	S	S	S	S	S	S	S	S	S
B929	S	S	S	S	S	S	S	S	S	S	S	S	S	S	S
B937	S	S	S	S	S	R	S	S	D	S	S	S	S	S	S
B938	S	S	S	S	S	R	S	S	D+	S	S	S	S	S	S
B939	S	S	S	S	S	S	S	S	S	S	S	S	S	S	S
B1014	S	S	S	S	S	S	S	S	S	S	S	S	S	S	S
B1198	S	S	S	S	S	R	S	S	D+	S	S	S	S	S	S
B1207	S	S	S	S	S	R	S	S	D	S	S	S	S	S	S
B1209	S	S	S	S	S	S	S	S	S	S	S	S	S	S	S
B1252	S	S	S	S	S	S	S	S	S	S	S	S	S	S	S
B1258	S	S	S	S	S	R	S	S	D	S	S	S	S	S	S
B1265	S	S	S	S	S	R	S	S	D	S	S	S	S	S	S
B1270	S	S	S	S	S	R	S	S	D	S	S	S	S	S	S

Note: fusidic acid (FD), cefoxitin (FOX), ceftaroline (CPT), ciprofloxacin (CIP), clindamycin (DA), chloramphenicol (C), erythromycin (E), gentamicin (CN), linezolid (LZD), quinupristin-dalfopristin (QD), rifampicin (RD), teicoplanin (TEC), tetracycline (TE), tigecycline (TGC), Vancomycin (VA). S – susceptible; R - Resistant All strains were susceptible to clindamycin but the table indicates the phenotype of inducible resistance on erythromycin resistant strains.
